# Perspectives on Palliative Care Approaches in People with Advanced COPD: A Qualitative Study of Patients Attending a Breathe Easy Clinic and Day Hospice

**DOI:** 10.5334/ijic.7748

**Published:** 2025-03-28

**Authors:** Barbara Gonçalves, Joanne Lusher, Audrey Cund, Caroline Sime, Eileen Harkess-Murphy

**Affiliations:** 1 University of the West of Scotland, UK; 2 Provost’s Group, Regent’s University London, UK; 3 Scottish Partnership for Palliative Care, UK

**Keywords:** COPD, palliative care, hospice care, peer group, self-help groups, day care

## Abstract

**Introduction:**

People with advanced chronic obstructive pulmonary disease (COPD) are well recognised to experience high levels of unaddressed physical and psychosocial symptom burden. Palliative care provides viable support that strives to relieve the sufferings and optimise quality of life for patients. This study aimed to identify factors that contribute to satisfaction and well-being of people with advanced COPD while attending services which offer palliative care approaches.

**Methods:**

A descriptive exploratory qualitative study using semi-structured interviews was conducted. Nineteen participants (67 ± 9 years) were recruited through Breathe Easy clinic (n = 13), and day hospice (n = 6) in the United Kingdom.

**Results:**

Both types of services were noted for bringing substantial contributions to patients’ lives. Healthcare professionals’ empathy and skilled communication were particularly important, with participants adjusting and accepting their limitations more easily when they understood their disease. Early introduction to services with palliative care approaches, along with referrals to support groups and education, helped normalise their experiences and improve symptom management.

**Conclusion:**

Providing psychological and educational interventions can lead to improvements in social aspects of patients’ lives or the development of self-management techniques to cope with the disease both physically and mentally. Early palliative care involvement is essential in this population.

## Introduction

Chronic obstructive pulmonary disease (COPD) is a preventable and treatable public health issue, which is estimated to rise in approaching decades due to population ageing and unceasing exposure to COPD risk factors, such as noxious gases or particles [1,2]. COPD is a lung condition characterised by chronic respiratory symptoms, such as dyspnoea, cough, and phlegm production, as a consequence of abnormalities of the airways which contribute to persistent airflow obstruction [2,3]. The COPD disease trajectory is progressive and debilitating, marked by alternating periods of deterioration and improvement in function, along with long phases of slowly declining health and acute worsening of respiratory symptoms, called exacerbations, which may cause a threat to life [4]. Hence, over time the focus of care shifts from life-prolonging therapies to palliative interventions aimed to alleviate symptoms and improve lung function and quality of life [5,6]. The trajectory of COPD is lifelong, and the onset of symptoms can be misleading and, therefore, unapparent [7]. Early detection of COPD is essential for preventing disease progression and clinical deterioration management [8]. Two-thirds of patients with COPD in the United Kingdom are not diagnosed in the early stages, and that late diagnosis puts people with COPD at higher risk, causing earlier first exacerbations and an increased incidence [9].

Over 10% of adults in need of palliative care suffer from chronic respiratory diseases [10]. While there is the availability of palliative care services in the United Kingdom, studies show that they are not effectively utilised for people with COPD [11,12,13]. Within healthcare, diverse services provide palliative care for the COPD population [14,15]. Moreover, palliative care services for people with COPD are organised differently in various regions of Europe [16]. In the United Kingdom, they can function as specialist services, exclusively providing palliative care [17,18], and non-specialist services, also known as generalist palliative care services, occasionally delivering palliative care [19]. Generalist palliative care is typically provided by primary care professionals and secondary care specialists treating people with life-threatening diseases who have fundamental palliative care skills and knowledge, with palliative care not being the focal point of their work [17]. Specialist palliative care can be organised in various formats, such as inpatient wards or consultation teams within hospitals or hospices, day care or home palliative care [20,21,22]. Moreover, integrated and multidisciplinary services such as breathlessness services, or Breathe Easy clinics, incorporate palliative care principles to aid people with chronic diseases in managing their breathlessness [23]. These services adopt different models and approaches to supportive care, operating across both levels of palliative care and are offered to patients with diverse conditions or focusing only on people with COPD [24,25,26]. Despite their diversity, all aim to improve the quality of life of people suffering from breathing problems associated with life-limiting illnesses [23,27,28].

Previous research has assessed the effects of palliative care on well-being and found that it did not improve their quality of life directly; nevertheless, it increased the number of people who chose advanced care [29,30]. This increase may suggest that palliative care offers more comprehensive support and information about care alternatives, as it usually involves detailed discussions about prognosis, goals of care, and treatment options. Thus, people receiving such care are likely to be better informed about their choices that align with their preferences and values.

Advanced COPD applies to a later stage of COPD when symptoms do not respond well to treatment and continue to get worse over time [31]. At this stage, patients may benefit from the early integration of palliative care due to its multi-professional person-centred and team-centred approach which may help advise people about the progress of the disease more effectively, enhancing their comprehension of the life situation, often perceived as hopeless, and helping them accept it [32]. The quality of contact with healthcare workers is another factor affecting the care of people with COPD. The conceptual relationship with healthcare providers has the potential to improve the care of people with chronic diseases, namely through better rapport between healthcare professionals and their patients, as well as the more active involvement of patients, leading to enhanced treatment compliance, which, in turn, can improve health outcomes [33,34,35].

It appears that there are opportunities within palliative care services which are beneficial to managing and improving the care of individuals living with COPD. However, there remains a lack of specific evidence about the needs and experiences of people with advanced COPD receiving palliative care services, whose role has yet to be thoroughly investigated. Moreover, this underrepresented population requires greater attention, as limited data exists on their participation in breathlessness services and day hospices [21,22,23,25,26,36].

The present study, therefore, aimed to understand perspectives on living life with advanced COPD and the experience of attending a Breathe Easy Clinic service and day hospice service.

## Methods

### Study design

The qualitative descriptive design was employed in this study, enabling a thorough account of the experiences and views of people living with advanced COPD and the appreciation of the subjective nature of the examined problem and the participants’ unique circumstances. Theis design allowed for the subjective nature of the investigated problem and the diverse difficulties of the participants, with results presented in order to reflect on the research aims [37].

This study adopted pragmatism as the most suitable paradigm for this research. Pragmatism draws on multiple ideas, including “what works” [38,39]. Accordingly, the research aims to direct the choice of methodology, instruments and data analysis. Therefore, collecting data from different services offering palliative care rather than from one was preferred, as it reflects the real-world and different pathways of care that people with COPD receive. This study adhered to the COREQ guideline for reporting qualitative research [40].

### Recruitment and procedures

The study was conducted in West and Central Scotland, United Kingdom. Purposive sampling was selected to focus on a specific population and to narrow the sample to individuals attending palliative care services and those with advanced COPD. Criteria were employed in the form of sampling chosen, which means that all selected participants needed to meet the predetermined criteria for quality assurance [39]. People with COPD attending a Breathe Easy clinic and day hospice services were screened by a designated individual from the healthcare team for eligibility criteria (Table 1).

**Table 1:** Inclusion and exclusion criteria.


INCLUSION CRITERIA	EXCLUSION CRITERIA

Classification of airflow limitation severity in COPD: Severe III GOLD (30% ≤ FEV1 < 50% pred.), Very severe IV GOLD (FEV1 < 30% predicted). Attended a supportive clinic or a day hospice at least 2 times. Speaking English.	Diagnosed psychiatric or cognitive disorders other than depression, anxiety and panic attacks. Diagnosed progressive neurological or neuromuscular disorders. Active cancer. Another uncontrolled or unstable disease. Any other respiratory disease.


In the Breathe Easy clinic, the respiratory consultants identified potential participants and informed them about the study. If patients expressed the willingness to participate, they had an opportunity to discuss further details with the first author, who was in the clinic on the same day. The potential participants received from the first author a Participant Information Sheet, as well as brief oral information about the study, and they could ask questions in case of any doubt. Subsequently, if patients initially showed interest in participation, phone numbers were collected with their permission to be contacted by the first author in the following days to check whether they wanted to proceed or not. In the day hospices, eligible participants were initially identified by the care team. They were approached directly by an appointed medical team member and received a Participant Information Sheet. Later, the appointed member contacted the first author, providing the phone numbers of the potential participants.

The Breathe Easy clinic in an ambulatory care hospital, in a large urban area, provided a disease-specific support group every month. The support groups were assisted by people with diverse severities of COPD and managed with the support of a local respiratory team. Patients had also opportunities for out-of-calendar consultations to discuss any concerns they may have had. Day hospice services were attended by patients with diverse malignant and non-malignant diseases for a 12-week programme, with some having a limitation of 2 people with oxygen therapy per group due to facilitates’ limitations of oxygen outlets and internal regulations. Breathe Easy clinic did not have this limitation. Both services offered information and advice on different aspects of a patient’s disease, including symptom management control and educational sessions involving looking after themselves and planning for the future and a chance to meet others in a similar situation. Moreover, they offered emotional support and complementary therapies run by expert volunteers and specialist programmes for people with breathing difficulties. Activities included gardening, gentle exercise, and crafts. In some cases, patients could make an appointment for a manual massage, while three hospice goers were able to access beauty treatments, including hairdressing and manicures. Also, participants were recruited from weekly pulmonary therapy sessions offered by a hospice.

### Participants characteristics

A total of nineteen people agreed to participate in this study. Out of the twenty-three people with advanced COPD in the Breathe Easy clinic who met the inclusion and exclusion criteria, thirteen chose to participate. This study involved collaboration with five hospices, three of which found eligible patients in their databases. One hospice recognised four patients, out of whom only one participated. The second participating hospice found two eligible patients who participated in this study. The third hospice identified five patients who matched the criteria, with three choosing to take part in the study. Table 2 presents characteristics of the participants.

**Table 2:** Characteristics of the participants.


CHARACTERISTICS	ALL PARTICIPANTS (n = 19)	BREATHE EASY CLINIC (n = 13)	DAY HOSPICE (n = 6)

**Gender** (male/female)	9/10	8/5	1/5

**Age** (mean, years)	67.3 ± 9.3	66.6 ± 6.5	68.67 ± 5.71

**Smoking status** (ex-smoker/smoker)	16/3	10/3	6/0

**Educational level**			

Secondary	13	8	5

Vocational	4	4	0

Higher	2	1	1

**Marital status**			

Married/partnership	10	9	3

Divorced/separated	3	3	0

Widowed	4	2	2

Single	2	2	1

**Antidepressants** (yes/no)	10/9	6/7	4/2

**Therapies during the disease course**			

Group pulmonary rehabilitation	13	10	3

Individual pulmonary rehabilitation	8	7	1

Self-management programme	8	8	0

Relaxation therapy	8	5	3

Self-help group	5	4	1

Counselling	3	3	0

Massage	3	2	1

No therapies	3	1	2

**Oxygen therapy** (yes/no)	12/7	7/6	5/1

**FEV 1% predicted** (n = 17)*	32.4 ± 8.1	30.2 ± 7.6	39.58 ± 2.52

**GOLD stage** (n = 17)*			

Severe	10	6	4

Very severe	7	7	0


*Missing data.

### Data collection

In-person, semi-structured interviews were carried out using an interview guide with open-ended questions by the first author, a qualified healthcare professional. The interviewer had no previous affiliation with the participants and no influence on their treatments. After agreeing to take part, the participants were met at the place and time chosen by them (either at their home, hospital or hospice premises) to ensure the most comfortable and appropriate place and time for them. For the interviews conducted in a clinical setting, the clinical team identified a suitable room which ensured the privacy needed for the meeting. Participants could choose to have a carer present, which occurred twice in the study when spouses accompanied male participants, offering additional information and support. No interviews were repeated.

Interviews were audio recorded and lasted 25–105 minutes, with an average time of 42 minutes. They were transcribed verbatim and only contained a name synonym; no individually identifiable data were recorded on the transcripts. Additionally, field notes were taken. The interview schedule incorporated key aspects, concentrating on the individuals’ general story of COPD and discussing the experience of everyday living with this disease in order to make them feel comfortable to facilitate the exploration of interview topics. Other areas centred on social life and relationships, psychological needs, experience and satisfaction from healthcare service, general needs of the patients and additional suggestions of the participants (Table 3). Participants were recruited until the discussed issues were fully understood and thematic data saturation was obtained [41,42].

**Table 3:** Interview Schedule.


Understanding the diagnosis and everyday life What a typical day is like for you living with COPD? What symptoms you experience during a normal day? What do you understand about your illness? Social life and relationships How would you describe your social life lately? How has the disease affected your social life (for example, contact with relatives, and friends)? What do you do to make yourself feel better? What do you think could be done in order to change and improve your social life? Psychological needs How would you describe your mood recently? How does living with COPD affect your mood and emotions? If you experience periods of anxiety, how do you manage them? Do you receive any psychological and social support? If yes, could you please describe what kind of support it is? How would you describe the support you receive and your satisfaction with it? Do you have some suggestions that could be made in order to change and improve your mood and emotions? Experience and satisfaction from the healthcare service Who helps you decide on the treatment and care you want to have? What decisions have you made regarding your treatment and care? What brought you to the hospice/Breathe Easy clinic? Could you please detail the process that led you to this service? What suggestions could you make to improve the process? What do you think about the service that is being provided to you? Is there any type of therapy or treatment that you feel that helps you the most to feel better? Which professionals do you consider most helpful? Needs of patients What do you feel is the most important factor in helping you to feel supported while living with COPD? How would you describe your satisfaction with the support provided by the service you attend? Do you feel you have been given enough information about your illness? If you had any questions about your disease, who would you think could answer them?


### Data analysis

Data analysis was carried out with NVivo 12 software. All interviews were analysed using Framework Analysis, which creates an organised model for managing and charting the data into a framework matrix and provides transparency [43]. The first author conducted coding and data analysis. Throughout, co-authors oversaw all processes of the analysis and provided independent scrutiny and feedback to guarantee reliability, reduce bias, and affirm the findings’ validity and rigour. Line-by-line coding was initially performed and as the coding process unfolded and themes began to emerge, an analytical framework gradually developed. A framework matrix was created to summarise the data, organising it by category and theme for each transcript. The relationships between these codes were examined across all transcripts, which helped in forming the themes. Themes were derived inductively. They were compared and reorganised by using the continuous comparison process during the review of data across the matrix. Manual tables were created to physically examine the associations among the concepts and typologies which evolved from them as well as relationships between the concepts. The participants did not provide feedback or corrections to the transcripts or findings.

## Findings

The analysis generated three key themes: attending palliative care across services, interaction and communication with healthcare professionals, and the value of having social connections (Table 4).

**Table 4:** Themes and subthemes.


THEME	SUBTHEMES

Attending palliative care across services	Experiences of the variety of offered support Beneficial aspects of received treatment

Interaction and communication with healthcare professionals	Empathic attitude of healthcare professionals Timeliness and accessibility of care Early education and patient empowerment Clear communication

Value of having social connections	Connection and a feeling of belonging Emotional comfort Motivational influence


### The role of multi-level care on patients’ experiences

Participants received palliative care support from different professionals in specialist services and services with integrated palliative care principles. Specialist palliative care was provided through day hospices and healthcare professionals and made a positive impression on the participants. One participant who had previously attended hospice day care, recalled the experience as relaxing and enjoyable, highlighting the emphatic hospice staff as a vital factor of their satisfaction with care: “It’s quite extraordinary to be within our kind of medical place that’s not in such a rush. There’s an incredible amount of kindness, a load of compassion which I find to be very reassuring” [Participant 10, female, 63 years, Breathe Easy clinic].

Integrated palliative care approach provided by the multidisciplinary respiratory team at the Breathe Easy clinic, was described as valuable by most participants. They especially appreciated the consultants’ good and timely treatment management, providing clear information and treatment choices. It was comforting for participants to know that they could have an extra, out-of-calendar consultation with respiratory consultants in the support clinic if they experienced any difficulties.

Another treatment which helped the participants was body massages, provided in a Breathe Easy clinic and in day hospices, which relaxed the patients and relieved muscle tension. Additionally, having received the massages, the participants felt pampered and it always lifted their mood. Relaxation sessions were another way to improve well-being and having these introduced early can help relieve tension and decrease anxiety: “It’s made me more peaceful, coming here. I love coming here because it’s a day away, and it’s about me, and I can get my nails done and my hair done, have my feet and my legs massaged, which is just absolutely wonderful and then I have a bit of relaxation. It’s lovely.” [Participants 15, Female, 73 years, day hospice].

Another additional support were formal social care workers who visited and helped two participants. They both stated that having formal carers took a burden off them and they felt less anxious and more supported. For some participants, however, having formal carers meant a perceived lack of control: “That would mean I’m not in control. Probably it would make it easier for somebody to come and dress me. But no, I couldn’t cope with that.” [Participant 6, Female, 62 years, Breathe Easy clinic].

### Empathic, timely care and effective communication as key contributions to care satisfaction

Having a reassuring and empathic leading doctor was of the utmost importance to many. Some felt they had an excellent rapport with their respiratory consultants and felt free to ask them any questions. They had the impression that they were fully informed about their condition, stage and possible further treatment, enabling them to trust their respiratory consultants: “I’ve been very fortunate that this new consultant that I’ve got, they’ve bent over backwards to try and make life a wee bit more bearable for me and I appreciate that.” [Participant 8, Female, 66 years, Breathe Easy clinic].

Being timely managed was an essential factor in improving satisfaction from the care received. Facilitations, such as contacting healthcare professionals, and having a phone number of a nurse or a GP, helped those who had this opportunity. However, three participants found it challenging to contact a GP surgery by phone and noted it as a barrier to a timely diagnosis of a pulmonary infection.

Most participants received home visits by respiratory or community nurses after being diagnosed, and almost all knew about the availability of home visits by nurses and how and whom to ask if they needed them. The participants felt that they could trust the nurses, communicate efficiently and ask them questions regarding treatment or the disease. Individuals who had routine home visits seemed more self-confident and more satisfied with the care they received: “The best thing I’ve ever had is the support workers. The nurses are brilliant. (They) explained everything to you. You’ve got a better understanding of what’s actually happening there because a GP doesn’t have time to sit and explain.” [Participant 3, Female, 63 years, Breathe Easy clinic].

Participants who tended to be more often admitted to hospitals reported a higher need for routine home visits from a nurse. Some participants would prefer to have received home visits from a nurse with management support and administration of medications at home rather than having to go to a hospital. They believed nurse home visits could help them avoid developing infections and unnecessary hospital admissions: “Just somebody to come up every so often because you can feel as if your chest infection isn’t only a chest infection and you say to yourself, it might just be a wee… you don’t feel well, then you leave it for two or three days and then it becomes too late.” [Participant 9, male, 68 years, Breathe Easy clinic].

People interviewed in this study valued and benefited from good communication and easy access to healthcare professionals, as early recognition of the problem was linked with more control over infections and hospitalisations. Good communication was connected with proper education, and openness to express patients’ doubts and complaints. Early education of the participants was noted as a contributing factor both to accepting the limitations caused by the condition and to better disease management. Knowing what to expect in the disease progression allowed the participants to acknowledge what would happen to them and helped them to minimalise psychological distress: “I know what caused it. I know that I’m not going to get any better, and it’s just going to be a deterioration over the months or years, however how long it takes. I understand what’s going to happen at the end, and I’m quite aware of it.” [Participant 8, Female, 66 years, Breathe Easy clinic]. Conversely, not understanding the diagnosis contributed to increased frustration: “I don’t know what’s causing that. I don’t know when it started. I don’t know, because I had always fresh and open air. And this is really a big shock. I had asthma and this whatever… (COPD) I don’t know where this came from.” [Participant 2, male, 85 years, day hospice].

### Benefits of social interaction and peer support in COPD management

The opportunity for socialising with peers was another beneficial factor that could be brought to the participants’ lives. Support groups were occasions to meet people with COPD or other debilitating diseases. Some participants felt that meetings with other people during support groups with a similar diagnosis could make up for the loss of their social life and was a motivation to leave home. Participants considered the hospice beneficial, as it allowed them to socialise. They felt it was a place where they could relax and enjoy time with people with similar problems: “It’s the only time you get to be yourself. You could be more relaxed with people because you are just in the same boat.” [Participant 16, Female, 64 years, day hospice].

Having the opportunity to talk to their peers dealing with similar struggles, participants could compare and exchange their experiences and observe how other people coped. Interacting with others who share the same challenges had a potential to help participants realise that they were not alone with their diagnosis, as other people were experiencing similar lifestyle limitations. Feeling a part of a community was comforting for many: “It introduced me to people who already had what I had and that helped. Knowing that you’re not the only person who has this (COPD). Because you could be sitting in this house and out there, you’d never meet anybody who has the same thing.” [Participant 22, male, 74 years, Breathe Easy clinic].

Finding new ways to enjoy their lifestyle within their limitations could be another positive consequence of acceptance. Being introduced at the beginning of the diagnosis to different people with similar experiences was of paramount importance in accepting their disease, forming adjustments, and developing coping mechanisms. Oftentimes, seeing people even in worse condition than theirs allowed them to realise that they were not the only ones having these problems, which was soothing to some: “I’m not the only one with this thing and there were people far worse than me.” [Participant 22, male, 74 years, Breathe Easy clinic].

The majority of participants interviewed in this study were recruited from a Breathe Easy clinic, which offered a disease-specific support group, while day hospices were attended by patients with a wide range of malignant and non-malignant diseases. However, it was noticed that patients who attended formal support groups with solely COPD, compared to those in day hospices, felt higher satisfaction concerning sharing experiences and a feeling of belonging, due to higher exposure to people with the same diagnosis. Nevertheless, both types of care were noted for bringing considerable positive contributions to patients’ lives. Overall, having contact with peer groups brought motivational, educational and social benefits. Both services played an important role in normalising patients’ experiences and enhancing self-management, consequently improving their mood.

## Discussion

People with COPD are vulnerable and experience high physical, psychosocial and spiritual burdens; therefore, identification of their needs in the early stages can help provide needs-based palliative care [44]. Regardless of the considerable burden of COPD, and recommendations of adequacy of palliative care to control symptom management and implement palliative care services in the practice, there are currently no specific guidelines for it [12,45]. Likewise, there are no established definitions or recommended frameworks for incorporating palliative care into respiratory services. This highlights the need for national guidelines that encourage early referrals to palliative care and integrate palliative care more effectively into COPD management [46].

In the United Kingdom, different integrated models of palliative care are implemented for individuals with COPD, including breathlessness services or specialist palliative care. To date, studies in the field have drawn data from a single level of care that found patients reported satisfaction with the service which stemmed mostly from elements such as personalised care, interpersonal skills of the staff, and personal education, as well as empowerment, improved mastery of breathlessness, boosted confidence and positively impacted emotional health [25,36,47,48]. These elements are reflected in this study with patients expressing improved confidence in managing their condition stressing the value of a comprehensive and empathetic approach to COPD care. Thus, personalised programmes addressing psychological, educational and self-management aspects, delivered from different levels of care, promise to improve disease management. This study further adds that people with COPD referred to palliative care and education in the early stages of their disease had more self-management skills compared to those who received palliative care only in the advanced stages of COPD. This links back to the importance of patient empowerment. As previously reported in a study with people with chronic pain, the multidisciplinary approaches can improve coping resources, and empower patients to manage emotionally related symptoms through self-regulatory, behavioural and cognitive techniques [49].

Lack of sympathy and empathy, unsatisfactory clarification or insufficient information and impolite communication with healthcare personnel are some of the most common complaints of patients and carers [50]. In this study we reported that when the participants trusted the healthcare professionals, they were more willing to express their complaints, knowing that they were heard and often addressed. Additionally, having an opportunity of regular contact with healthcare professionals gave them a feeling of being regularly tended to and not left alone. Moreover, the results of this study match those observed in earlier studies with chronic disease patients and indicate that adequate communication skills are crucial to building a good rapport between patients and healthcare professionals, leading to improved patient empowerment [33,34]. Patient participation and the doctor-patient relationship are crucial and it is suggested that healthcare personnel spending more time with patients trying to build a trusting relationship and provide education could be beneficial [33,34]. The findings from this study suggest good information about the disease led to high involvement of patients in their treatment. This could be attributed to the fact that most patients attended support groups and educational sessions in their community service or via supportive care clinics, which suggests that a proper understanding of the diagnosis empowered patients to be actively involved in their treatment and develop self-management skills.

Previously authors have reported the importance of patient satisfaction in communication with physicians [51,52]. Moreover, a study with patients with mild and moderate COPD stated that healthcare professionals should realise that the way the diagnosis is communicated and disclosed evidently affects patient’s perception of the disease [53]. This also coincides with observations of this study, which confirmed that proper communication in the early stages of the disease paved the way for education about the diagnosis and was a factor which facilitated their acceptance of the disease. Further, it indicated that proper communication could lead to referrals for other required treatments and activities, which in turn, could directly improve patients’ symptoms and their coping with the condition. Hence, good communication is key to ascertaining if patients linger in negotiation or progress towards acceptance and new understanding.

In this study, the participants having a high level of understanding of their condition and level of treatment, often linked it to the exemplary communication skills of their pulmonologists and nurses and consequently felt they were in control of their care. However, those who complained about a lack of dialogue and information from their healthcare professionals had certain shortcomings in terms of control over their treatment. Current evidence suggests a concerning lack of COPD-specific information being either shared or retained by people living with COPD. Despite attending specialist palliative care, many individuals with COPD reported a troubling lack of information about their disease, its course and options of treatment and care [21]. Also, patients not regarding themselves as sick further hampered the identification of their needs due to a lack of understanding of their disease and its symptoms [22,54]. Patients’ limited knowledge concerning the cause, process, and incurability of COPD was linked with physicians’ lack of ability to communicate with patients effectively [32]. Notwithstanding the acknowledged role of dialogue in palliative care, proper communication in this population remains an issue, and insufficient training in palliative care among pulmonologists is identified as a barrier to adequate communication with patients [55].

The present study showed that attending peer support with educational session groups from both types of services, Breathe Easy Clinic and day hospice, brought positive outcomes, both social and psychological. Participants attending day hospice and support groups regarded meetings with other people with similar problems as a social asset. It empowered them to leave their home, which in turn, allowed them to socialise, compare their disease experience with other patients, and normalise it. Evidence previously has acknowledged that peer support provides an opportunity for sharing and validating patients’ experiences with others [53,56]. Furthermore, this study agrees with prior research suggesting that day hospice support group could be a feasible solution for meeting patients’ needs, improving the well-being of people with COPD, enhancing their mood and self-esteem and avoiding feeling alone with their burden considering that they could share their experiences about their ill-health, and knowledge about the disease and empathically listen to one another’s problems, diminishing their social isolation [21]. This is further supported by wider literature in the field, which indicates that social support via breathlessness service can be a valuable component of early palliative care for people with COPD and improve their quality of life [23].

People with COPD are underserved in specialist palliative care and more research is required about people attending this service. Further investigations are needed to estimate the possible impact of non-pharmacological interventions recognised by GOLD [2], such as counselling, self-help groups, self-management programmes, relaxation and music therapy for people with advanced COPD attending palliative care services.

### Strengths and limitations

This study was conducted in the underrepresented population of people with COPD being the first study, to our knowledge, to recruit patients from hospices and a Breathe Easy clinic. By not limiting our recruitment to a single type of care, we were able to obtain more comprehensive and real-world results, allowing for a more diverse and representative view of this vulnerable population. Although the majority of participants attended Breathe Easy clinic at the time of data collection, this study offered a diversity of services and a broader spectrum of experiences, enhancing the applicability of the findings to practical solutions and potentially yielding more robust and generalisable findings.

Participation was voluntary; thus, the presented sample could be a group of patients less affected physically and psychologically. Although all participants attended services with integrated palliative care principles, some other patients, who were in poorer health, in more deteriorating condition or immobile, could not have been interested in participating in the study as it could have been too big an effort for them, either psychologically or physically. Hence, the findings cannot be generalised to all patients with advanced COPD. Moreover, this sample represented a limited group of participants from specific settings, and this could introduce standpoint bias. Therefore, these views may not be shared by other patients in different locations and services, and the findings may not represent views of people who receive care from other types of services.

## Conclusion

Key findings from this study have shown that empathy from healthcare providers, as well as education, facilitate the development of self-management strategies. The empathy of healthcare providers and support workers makes patients feel better understood, which in turn can reduce feelings of psychological distress. The timeframe in which participants received initial support from palliative care seems to play a crucial role in developing self-management strategies. Despite the positive aspects of palliative care intervention in advanced stages, it may be assumed that adding new therapies early in the diagnosis would bring more therapeutic benefits than introducing them in the advanced stage of COPD, allowing participants to learn techniques to cope with challenges as the disease progressed. Palliative care can be offered through different types of services, but a takeaway point from this study is that it is not important where the source of care comes from; any support brings varying but considerable positive contributions to the lives of people with advanced COPD.
